# A novel *HRAS* c.466C>T p.(Phe156Leu) variant in two patients with attenuated features of Costello syndrome

**DOI:** 10.1038/s41431-022-01139-1

**Published:** 2022-06-29

**Authors:** Suzanna Lindsey-Temple, Matt Edwards, Verena Rickassel, Theresa Nauth, Georg Rosenberger

**Affiliations:** 1 Department of Clinical Genetics, Liverpool Hospital, Sydney, NSW Australia; 2 School of Women’s and Children’s Health, Faculty of Medicine and Health, UNSW, Sydney, NSW Australia; 3 Paediatrics, School of Medicine, Western Sydney University, Hunter Genetics, Newcastle, NSW Australia; 4 Institute of Human Genetics, University Medical Center Hamburg-Eppendorf, Hamburg, Germany

**Keywords:** Genetics research, Disease genetics

## Abstract

Costello syndrome (CS) is caused by heterozygous *HRAS* germline mutations. Most patients share the HRAS variant p.Gly12Ser that is associated with a typical, homogeneous phenotype. Rarer pathogenic HRAS variants (e.g., p.Thr56Ile) were identified in individuals with attenuated CS phenotypes. The obvious phenotypical variability reflects different dysfunctional consequences of distinct HRAS variants. We report on two boys with the novel *de novo HRAS* variant c.466 C > T p.(Phe156Leu). Both had severe feeding difficulties, airway obstruction and developmental delay, which are typical findings in CS. They showed subtle facial and dermatologic features consistent with attenuated CS. They significantly differed in their musculoskeletal, cardiovascular and endocrinologic manifestations underscoring the clinical variability of individuals with identical, in particular rarer pathogenic HRAS variants. Functional studies revealed enhanced effector-binding, increased downstream signaling activation and impaired growth factor-induced signaling dynamics in cells expressing HRAS^Phe156Leu^. Our data further illustrate the molecular and phenotypic variability of CS.

## Introduction

RASopathies are syndromic conditions that result from germline alterations in genes coding for RAS pathway signaling proteins. Missense variants in the proto-oncogene *HRAS* underlie the RASopathy Costello syndrome (CS). Individuals with CS are predisposed to cancer and may have distinctive craniofacial features, cardiac anomalies, growth and developmental delays, as well as dermatological, orthopedic, ocular, and neurological issues [1]. Approximately 80% of CS-causing *HRAS* gene variants result in a p.Gly12Ser missense change and this variant is associated with the classic CS phenotype [1]. A more variable, milder or “attenuated” phenotype occurs with rarer pathogenic HRAS variants, such as p.Thr58Ile, p.Gly60Asp/Val or p.Ala146Thr/Val/Pro [1, 2].

Disease-associated changes of HRAS amino acid 12 or 13 impair intrinsic GTPase activity, confer resistance to GAPs and, thereby, trap HRAS in its active state independently from incoming signals. This results in increased activation of HRAS downstream signaling pathways, a functional consequence that is widely used to explain the pathobiology of CS. However, several studies demonstrated a more diverse spectrum of molecular defects of CS-associated HRAS variants, which result in dysregulated HRAS-dependent signaling dynamics [3–7].

Here, we aimed to characterize the clinical manifestations and functional consequences associated with the pathogenic *HRAS* variant c.466 C > T p.(Phe156Leu) and to compare our data to those for previously described CS-related *HRAS* variants.

## Materials and methods

The patients’ parents provided written informed consent for the participation in the study, clinical data and specimen collection and genetic analysis according to the Declaration of Helsinki and national legal regulations. They signed informed consent regarding publishing their data and photographs.

A description of the laboratory methods is given in the Supporting Information.

## Results

### Clinical summaries

**Fig. 1:**
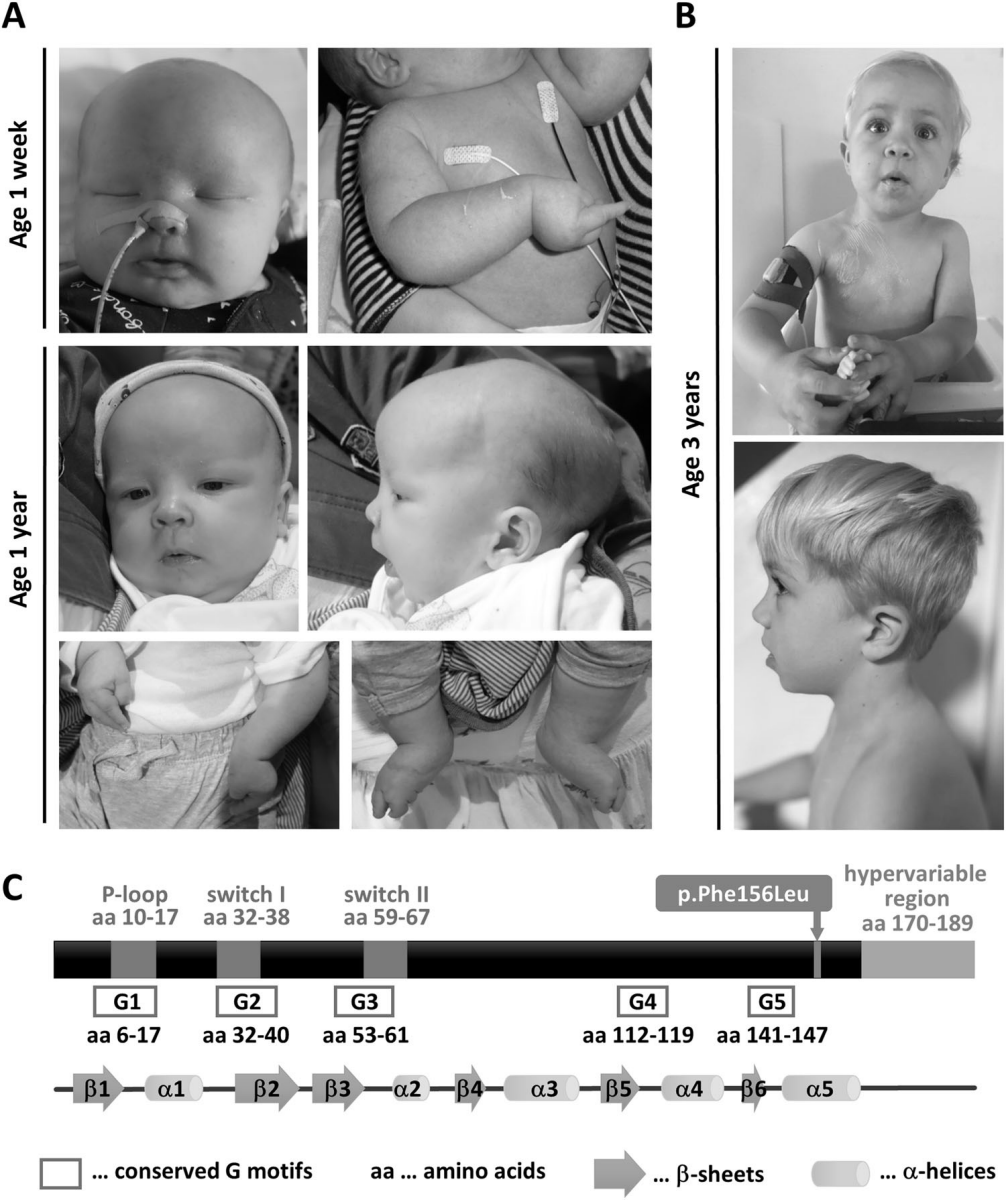
Photographs of patients 1 and 2. **A** Patient 1. Note macrocephaly, the adducted thumbs and wrist varus posture and congenital vertical talus. **B**. Patient 2. Note the prominent forehead, long philtrum and full cheeks. **C** Schematic representation of HRAS and position of the p.Phe156Leu variant.

*Patient 1*. The pregnancy was complicated by maternal gestational diabetes, polyhydramnios, fetal overgrowth and caesarean section (37 weeks). Birth weight was above the 97th centile, length was on the 75th centile, and head circumference was greater than the 98th centile (Table 1). Limb shortening, varus posture of wrist joints, adducted thumbs, elbow contractures, rocker bottom feet, extended knees, hip dysplasia and macrocephaly were noted (Fig. 1A). At 5 weeks old the boy showed recurrent severe obstructive apneic episodes with cyanosis, loss of responsiveness, seizures and choking episodes during feeding. Pharyngeal and laryngeal obstruction required supraglottoplasty and recurrent apneic episodes and aspiration required permanent tracheostomy. Dysfunctional swallowing and feeding difficulties were noted. Weight gains improved with small frequent percutaneous endoscopic gastrostomy feeds. Details on tonic clonic seizures and treatment are given in the Supporting Results. Abdominal ultrasound detected no malignancy. The patient was following visually, smiling and breathing normally. Tracheostomy was removed at 9 months old. He had generalized developmental delay, was unable to roll or sit with marked head lag. Deep palmar and plantar creases were noted. At 2 2/3 years, weight and length were below the 3rd centile and head circumference was greater than the 95th centile (Table 1). Cardiac arrhythmia was documented, seizures persisted. Trio exome sequencing identified two likely pathogenic de novo variants, c.466 T > C p.(Phe156Leu) in *HRAS* (NM_005343.4) and c.4907 G > C p.(Arg1636Pro) in *SCN1A* (NM_001165963.4). The latter most probably underlies the seizures in this patient (OMIM *182389).

**Table 1:** Comparison of the clinical presentation of patients with different HRAS variants.

HRAS variant	p.Phe156Leu		p.Ala146Pro/Val/Thr	p.Thr58Ile	p.Gly12Ser
Patient/number of probands References	Patient 1 This report	Patient 2 This report	*N* = 3 Gripp (2019), Chiu (2017), Zampino (2007), Gripp (2008)	*N* = 4 Gripp (2019), Gripp (2017), Gripp (2012), Gripp (2008), Hippala (2016)	*N* = 30 Gripp (2019), Gripp (2017)
Prenatal findings	GDM, polyhydramnios, large fetal size, Ces. S. 37 wks. gestation	Polyhydramnios, NVD Term	Uneventful, N/R	Borderline polyhydramnios (1/2)	Polyhydramnios (almost universal), large fetal size (frequent)
[*age*] Weight Length OFC	[Birth] 4.51 kg (>97^th^ ctl) 50 cm (75^th^ ctl) 41 cm (>98^th^ ctl) [2 yrs 8 mths] 10 kg (<3^rd^ ctl) 79.1 cm (<3^rd^ ctl) 52 cm (>95^th^ ctl)	[Birth] 3.5 kg (50^th^ ctl) N/D 36 cm (50^th^ ctl) [6 yrs 8 mths] 22.5 kg (50^th^ ctl) 113 cm (10^th^ ctl) 55.3 cm (>97^th^ ctl)	[Birth] Normal (3/3) Normal (1) Normal (2/2) [6 mths–6 yrs] < 3rd ctl (2/2) Normal (2/2) <3rd ctl (2/3)	Height < −2 SD below mean of age (0/4)	Height < −2 SD below mean of age (26/30)
Craniofacial features	Macrocephaly, short nasal tip, long philtrum, subtle dysmorphic features	Prominent forehead, long philtrum, full cheeks, macroglossia, subtle dysmorphic features	Microcephaly (1/3), flat occiput (1/3), low set ears (1), short nasal tip (2/2), long philtrum (2/2), mildly prominent earlobes (1), subtle dysmorphic features (2/2)	Macrocephaly (3/4), full lips (1/3), prominent ear lobes (2/4), subtle/mild dysmorphic features (3/4)	Macrocephaly (9/30), coarse facial features (universal), full lips (almost universal), large mouth (almost universal)
Musculoskeletal manifestations	Limb shortening, varus posture of wrist joints, adducted thumbs, elbow contractures, congenital vertical talus, extended knees, hip dysplasia	Tight Achilles tendons, gait abnormalities	Osteopenia (1), osteoporosis (1), hypotonia (1), coxa valga (1), minor involvement of joints (1)	Ligamentous laxity (2/3), hypotonia (1/2), mild pectus excavatum (1), narrow shoulders (1), limited elbow extension (1), cubitus valgus (1), limited supination (1), joint pain (1)	Ulnar deviation (24/30), hypotonia (22/30), tight achilles tendons (almost universal)
Cardiovascular abnormalities	Cardiac arrhythmias (ectopic beats)	HCM, VH, mild PS	HCM (2/2), tachycardia (1), VSD (1), PDA (1), MR (1), mild AR (1)	HCM (2/4), DCRV (1), MVP (1), moderate LVH (1)	HCM (13/30), PS (15–20%), ASD (5–7%), atrial arrhythmias (>50%)
Dermatologic findings	Mildly deep palmar and plantar creases	Thickened skin on elbows, excessive sweating, sensitive skin	Frontal bossing (1), sparse, short hair (1), long eyelashes (1), deep set eyes (1), dry skin (1), mildly deep palmar creases (1), thin, sparse, not curly hair (1), minor involvement of skin (1)	(Moderately) deep palmar/plantar creases (2/3), prominent digital pads (1), redundant soft tissue (2), fine hair (2/3), long eyelashes (1)	Deep palmar or plantar creases (universal), curly or sparse, fine hair (almost universal)
Neurologic and behavior issues	LOR, tonic clonic seizures	Mild ventriculomegaly, (no seizures)	Arachnoid cyst (1), mild CTH (1), anxiety (1)	Possible ADHD (1), tremors (1), behavior problems (1), very active (1), poor fine motor skills (1), anxiety (1), tonic-clonic seizures (1)	Sleep disturbance (frequent), anxiety (sometimes), behavior problems (50%)
Neurocognitive findings	DD, little active movement, no communication	Moderate DD	(Moderate) DD (2/2), moderate ID (1)	DD (1/3)	DD (universal)
Gastroenterologic, endocrinologic and metabolic findings	dysfunctional swallowing, FTT, FTP, choking episodes	FTT, FTP, severe GORD, severe hypoglycemia, hyperinsulinism, cow’s milk intolerance	(Severe) FTT (3/3), FTP (2/3), metabolic acidosis (1), GORD (1), GH deficiency (1), swallowing difficulty (1)	FTT (3/4), FTP (2/3), pyloric stenosis (1/3)	FTT (universal), FTP (almost universal), GORD (frequent), hypoglycemia (at risk)
Hematologic and oncologic findings	No	No	No (2/2)	Papillomata (0/4), malignant tumor (0/4)	Papillomata (14/30), malignant tumor (2/30)
Respiratory and otolaryngologic findings	Obstructive apnea, aspiration, pharyngeal and laryngeal obstruction	Laryngomalacia	N/R	N/R	sleep apnea (at risk)
Other findings	No	Esotropia to right eye, (no hernia)	Inguinal hernia (1), Strabismus (1)	Strabismus (3/4), Nystagmus (1/4), severe myopia (1/4)	Strabismus (>50%), Nystagmus (13/30)

*GDM* maternal gestational diabetes, *Ces. S.* Cesarean section, NVD Term natural vaginal delivery, *OFC* occipitofrontal circumference, *ctl* centile, *HCM* hypertrophic cardiomyopathy, *(L)VH* (left) ventricular hypertrophy, *PS* pulmonic stenosis, *VSD* ventricular septal defect, *PDA* patent ductus arteriosus, *MR* mitral regurgitation, *AR* aortic regurgitation, *DCRV* double-chambered right ventricle, *MVP* mitral valve prolapse, *ASD* atrial septal defect, *LOR* loss of responsiveness, *CTH* cerebellar tonsillar herniation, *ADHD* attention deficit hyperactivity disorder, *DD* developmental delay, *ID* intellectual disability, *FTT* failure-to-thrive, *FTP* feeding tube placement, *GORD* gastroesophageal reflux disorder, *GH* growth hormone, *wks* weeks, *yrs* years, *N/D* not documented, *N/R* not reported.

Summaries are given for the variants p.Ala146Pro/Val/Thr, p.Thr58Ile and p.Gly12Ser: findings present/patients with information on the finding; (*n*), feature was reported in *n* individuals. Other findings include dental, oral, ophthalmologic and genitourinary manifestations.

*Patient 2*. was recently mentioned [1]. The boy’s weigh and head circumference were on the 50th centile after a pregnancy complicated by polyhydramnios (Table 1). Bilious vomiting, hypoglycemia, macroglossia and poor feeding were noted on day 1 of life. Daily episodes of hypoglycemia associated with hyperinsulinism were treated with diazoxide and chlorothiazide. Severe hypoglycemia persisted with bolus feeds and continuous feeds were given via gastrostomy, with elemental formula due to cow’s milk protein intolerance. Laryngomalacia was diagnosed by endoscopy and echocardiography identified hypertrophy of the ventricular myocardium and mild thickening of the pulmonary valve. Metabolic/endocrinological investigations did not yield a diagnosis for the atypical hyperinsulinism. Glucose levels were unresponsive to octreotide, were more stable on diazoxide and improved with prednisone suggesting increased insulin sensitivity in addition to hyperinsulinism. Subtotal pancreatectomy was required at 8 months old. During a 5 months hospitalization hypertrophic cardiomyopathy and severe gastroesophageal reflux were diagnosed. The boy has subtle CS facial features with a prominent forehead, long philtrum and full cheeks (Fig. 1B). He has tight Achilles tendons and shows gait abnormalities. Dermatologic findings included thickened skin on elbows, excessive sweating, and sensitive skin. The patient has a mild ventriculomegaly and moderate global developmental delay. Unilateral strabismus (esotropia) of the right eye was noted. At 6 2/3 yrs old hypoglycaemia episodes were still significant with several low glucose episodes repeatedly per day caused by any form of excitement or stimulation. Trio exome sequencing identified the *de novo HRAS* c.466 C > T p.(Phe156Leu) variant.

### Functional characterization

**Fig. 2:**
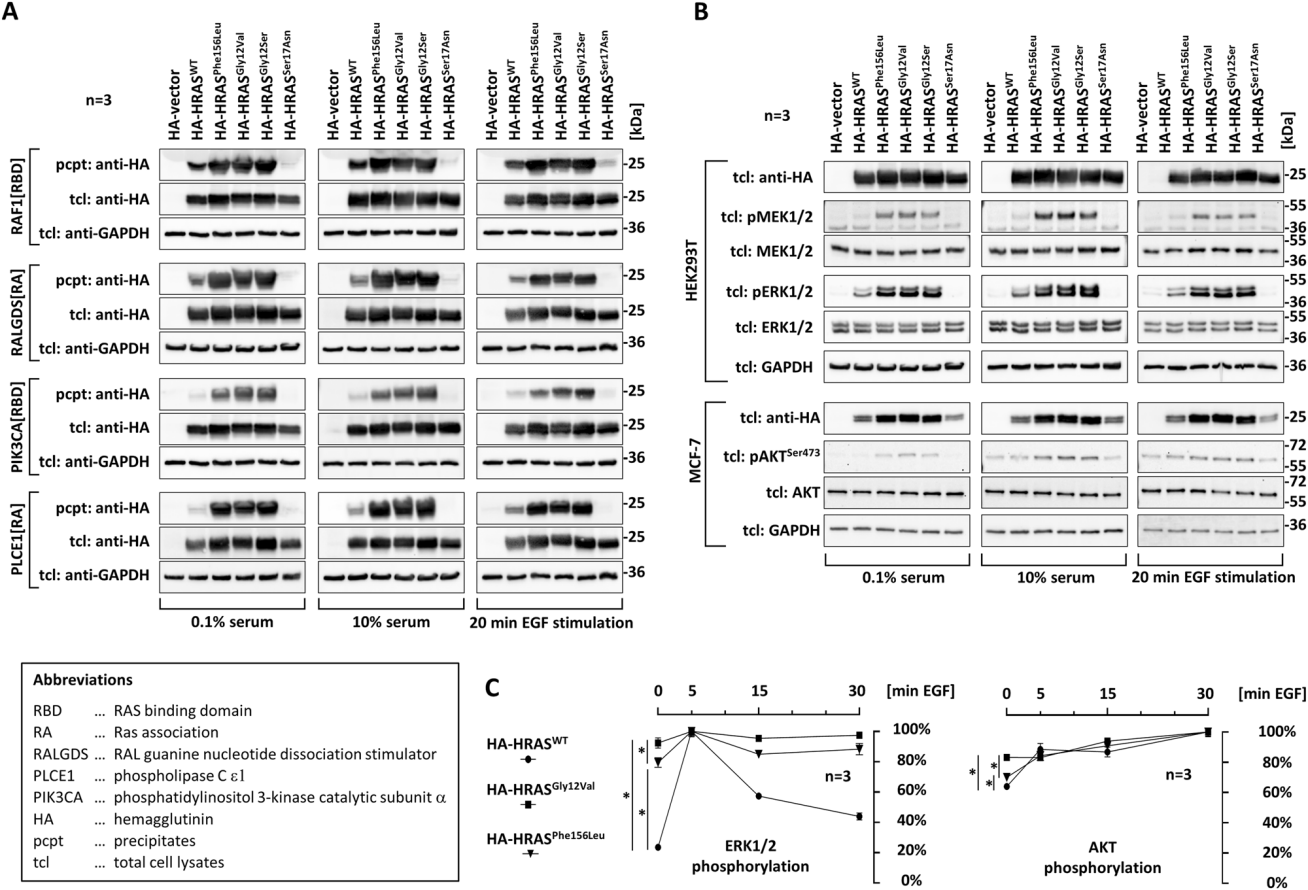
Functional consequences of HRAS p.Phe156Leu. **A** HRAS^Phe156Leu^ co-precipitates with RAF1, RALGDS and PLCE1, PIK3CA. HRAS variants were expressed in HEK293T cells under serum-starved condition, normal growth condition, or serum-starved condition followed by 20 min EGF stimulation. HA-HRAS was precipitated from extracts by using GST-fused effector peptides and subjected to immunoblotting. **B** Expression of HRAS^Phe156Leu^ enhances downstream signaling. HRAS variants were expressed in HEK293T and MCF-7 cells. Cells were cultured under serum-starved, basal and stimulated conditions. Lysates were subjected to immunoblotting. **C** Expression of HRAS^Phe156Leu^ impairs epidermal growth factor sensitivity. HEK293T cells transiently expressing HRAS^WT^, HRAS^Gly12Val^ or HRAS^Phe156Leu^ were stimulated with EGF for various times (5, 15, 30 min) or left untreated (0 min) and phosphorylation levels were determined by immunoblotting (Fig. S3). Values are relative to maximum levels and represent the mean of three experiments; for untreated cells significance levels are specified between data points [∗, *P* < 0.05; (two-tailed *t* test)].

The strictly conserved HRAS amino acid Phe^156^ is not located within a distinct functional protein motif (Fig. 1C) [8]. However, it’s part of the α5-helix that is critical for overall HRAS structure [8]. Our functional studies support pathogenicity of the HRAS p.Phe156Leu variant: We used GST-fusion proteins of interacting motifs from RAS effectors RAF1, RALGDS, PIK3CA and PLCE1 and precipitated activated HA-tagged HRAS protein variants from cell extracts (Fig. 2A). Oncogenic HA-HRAS^Gly12Val^ and CS-typical HA-HRAS^Gly12Ser^, but not the dominant-negative variant HA-HRAS^Ser17Asn^ strongly co-precipitated with any effector under any culture condition tested (Fig. 2A). The amount of HA-HRAS^Phe156Leu^ was elevated compared to HA-HRAS^WT^ in all precipitates. These data suggest that HA-HRAS^Phe156Leu^ accumulates in the active form and forms stable complexes with effectors.

By analyzing binding between HA-HRAS^Phe156Leu^ and the RAS-specific NF1 GTPase activating protein (GAP), we detected moderately enhanced GAP binding efficiency compared to HA-HRAS^WT^; however, this increase was less pronounced than observed for HA-HRAS^Gly12Ser^ and HA-HRAS^Gly12Val^ (Supporting Results, Figure S1). This suggests that p.Phe156Leu does not negatively interfere with NF1 GAP binding.

To gain insight into consequences of p.Phe156Leu on signal traffic, we measured levels of phosphorylated MEK1/2, ERK1/2 and AKT (Fig. 2B). Expression of HA-HRAS^Gly12Val^ or HA-HRAS^Gly12Ser^ but not HA-HRAS^Ser17Asn^ promoted MEK1/2 and ERK1/2 phosphorylation in HEK293T cells under any cell culture condition. Likewise, HA-HRAS^Phe156Leu^ enhanced phosphorylation of MEK1/2 and ERK1/2 compared to HA-HRAS^WT^ (Fig. 2B). AKT phosphorylation was marginally increased in HEK293T cells expressing HA-HRAS^Phe156Leu^, HA-HRAS^Gly12Val^ or HA-HRAS^Gly12Ser^ (Supporting Results, Fig. S2); however, HA-HRAS^Phe156Leu^, HA-HRAS^Gly12Val^ or HA-HRAS^Gly12Ser^ induced stronger AKT phosphorylation than HA-HRAS^WT^ in MCF-7 cells (Fig. 2B). Our data suggest that the p.Phe156Leu change intensifies HRAS downstream signal flux.

Impaired signaling dynamics rather than a simple static hyperactivation of RAS-dependent signaling may underlie the development of CS [3, 4]. We compared the intensity of EGF-induced signaling over time. EGF addition induced a strong ERK1/2 phosphorylation response in HA-HRAS^WT^ cells after 5 min followed by a decrease after 15 to 30 min (Figs. S3 and 2C). In contrast, cells expressing HA-HRAS^Phe156Leu^ or HA-HRAS^Gly12Val^ showed enhanced basal ERK1/2 phosphorylation (0 min EGF) and little further increase (Figs. S3 and 2C). Similarly, AKT phosphorylation was slightly stimulated upon EGF treatment in cells expressing HA-HRAS^WT^ but not or marginally in cells expressing HA-HRAS^Gly12Val^ and HA-HRAS^Phe156Leu^, respectively (Figs. S3 and 2C). These data suggest that the HRAS p.Phe156Leu variant impairs EGF-induced signal transduction efficiency within cells.

## Discussion

### Phenotype associated with *HRAS* c.466 T > C p.(Phe156Leu)

Our patients presented with prenatal polyhydramnios, severe feeding difficulties, musculoskeletal manifestations as well as gastroenterologic, endocrinologic and metabolic findings, which are common or frequent findings associated with both the typical CS-associated p.Gly12Ser variant and rarer HRAS variants (Table 1) [1–3, 7, 9–16]. Hyperinsulinism and hypoglycemia have been repeatedly reported in individuals with CS;[1, 5] however, in patient 2 these findings are uncommonly severe in terms of clinical presentation, management challenge and durability. Respiratory and otolaryngologic findings as well as cardiovascular abnormalities have also been described in patients with CS (Table 1) [1]. Notably, airway problems requiring tracheostomy has been reported as rare complication in patients with CS and different *HRAS* variants;[1, 14, 17] thus, this finding is not related to specific (rare or common) variants. On the other hand, both individuals showed milder facial features than patients with HRAS p.Gly12Ser; this is more in keeping with findings seen in individuals with rare *HRAS* variants affecting residues Thr^58^, Gly^60^, Lys^117^ or Ala^146^ (Table 1) [1–3, 5, 7, 9–11, 18]. The same is true for the dermatologic findings in both children (Table 1); however, since the skin phenotype varies with age, a final assessment should only be made in adulthood. Body length and height was in a normal range in patient 2, which has also been reported for individuals with HRAS variants p.Thr58Ile, p.Gly60Asp and p.Gly13Cys (Table 1) [5, 7, 10, 19]. Overall, the phenotypic presentation of the individuals described here may be best classified as less severe, with the exception of otolaryngology involvement, attenuated CS similar to that previously described for rare pathogenic *HRAS* variants [1–3, 5, 7, 9–11, 18].

### Functional consequences of HRAS p.Phe156Leu

Our data demonstrate that p.Phe156Leu results in the accumulation of active HRAS and dysregulated HRAS-dependent signaling dynamics. Phe^156^ is located in the α5-helix within the hydrophobic core of HRAS and, hence, it has a crucial role in its structural stability [8]. Structural considerations suggested that substitution of HRAS Phe^156^ by leucine affects contacts with surrounding residues and changes intrinsic functions of HRAS [20]. Indeed, p.Phe156Leu induced major structural changes, weakened HRAS contacts with Mg^2+^ and guanine nucleotides and increased GDP/GTP dissociation rates and levels of GTP-bound HRAS [8]. This is in contrast to the typical CS-associated HRAS p.G12S variant that perturbs GTPase activity. The germline variant p.Phe156Leu has also been identified in *KRAS* of patients with RASopathy and functional consequences of KRAS^Phe156Leu^ were determined (Supporting Discussion). Summarizing available data, the main molecular defect of HRAS^Phe156Leu^ is a strong acceleration of nucleotide exchange, but interaction with effectors and regulators is still functional. We conclude that *HRAS* c.466 T > C p.(Phe156Leu) is pathogenic. The p.Phe156Leu variant reduces growth factor sensitivity of HRAS, which is detectable as decreased stimulus-dependent increment of downstream signaling pathway activation. This is a common finding for CS-associated HRAS variants and underscores that impaired signaling dynamics is the central pathomechanism for CS (and related RASopathies) [3, 4].

## Supplementary information

Supporting Information

## Data Availability

All data generated or analysed during this study are included in this published article [and its supplementary information files]. The variant and the associated phenotype have been documented in the ClinVar database (Accession: VCV001327492.1; Variation ID: 1327492; Submission ID: SUB10779819).
