# Supplementary material for: A novel HRAS c.466C>T p.(Phe156Leu) variant in two patients with attenuated features of Costello syndrome
Source: Eur J Hum Genet. 2022 Jun 29;30(9):1088–93. doi: 10.1038/s41431-022-01139-1 (PMC9437031; doi:10.1038/s41431-022-01139-1)
Supplement: Supplementary file 1 — Supporting Information [file 41431_2022_1139_MOESM1_ESM.pdf]

SUPPORTING INFORMATION

A novel *HRAS* c.466C>T p.(Phe156Leu) variant in two patients with attenuated features of Costello syndrome

Suzanna Lindsey-Temple[1,2], Matt Edwards[3], Verena Rickassel[4], Theresa Nauth[4], Georg Rosenberger[4]

[1]Department of Clinical Genetics, Liverpool Hospital, Sydney, NSW, Australia

[2]School of Women's and Children's Health, Faculty of Medicine and Health, UNSW, Sydney, NSW, Australia

[3]Paediatrics, School of Medicine, Western Sydney University, Hunter Genetics, Newcastle, Australia

[4]Institute of Human Genetics, University Medical Center Hamburg-Eppendorf, Hamburg, Germany

SHORT TITLE: *HRAS* c.466C>T p.(Phe156Leu) causes Costello syndrome

CORRESPONDING AUTHOR

Georg Rosenberger, MSc, PhD

ORCID 0000-0003-3625-3109

Phone +49-40-741054534

Email rosenberger@uke.de

SUPPORTING MATERIAL AND METHODS

Plasmids

We amplified the coding region of wild-type *HRAS* to generate an expression construct by using HRAS-specific PCR primers and *HRAS* cDNA as a template. Mutated *HRAS* cDNA inserts (c.34G>A [p.Gly12Ser], c.35G>T [p.Gly12Val], c.50G>A [p.Ser17Asn] and c.466C>T, [p.Phe156Leu]) were established by PCR-mediated mutagenesis. Purified PCR products were cloned into pENTR/D-TOPO (Invitrogen, Karlsruhe, Germany) according to the provided protocol. Constructs were sequenced for integrity and used for subcloning wild-type and mutated *HRAS* coding regions into plasmid pMT2SM-HA-DEST (N-terminal HA epitope). $NF1^{333}$ in pGEX-4T3 encodes residues 1,198–1,530 of human NF1 comprising the functional GAP-related domain that is able to bind to wild-type HRAS and stimulate GTP hydrolysis.

Cell Culture and Transfection

HEK293T (derived from human embryonic kidney cells) and MCF-7 (breast cancer cell line) cells were cultured in Dulbecco's Modified Eagle Medium (DMEM; Invitrogen) containing 10% serum (Invitrogen) and penicillin-streptomycin (100 U/ml and 100 mg/ml, respectively) (Invitrogen) at 37°C and 5% CO2. Transfections were performed using Lipofectamine 2,000 Reagent (Invitrogen) according to the manufacturer's protocol. For experiments, cells were cultured under serum-starved condition (0.1% serum), normal growth condition (10% serum), or cells were blocked overnight by serum starvation (0.1% serum), followed by incubation in DMEM containing 10 ng/ml EGF (Sigma, Taufkirchen, Germany).

Pull-Down Assays

The RAS-binding domain (RBD) of RAF1 (amino acids 51–131), the RBD of PI3K (PIK3CA) (amino acids 127–314), the RAS association (RA) domain of RALGDS (amino acids 777–872) and the RA domain of PLC1 (PLCE1) (amino acids 2,130–2,240) were used to specifically precipitate GTP-bound RAS proteins from cell extracts. Preparation of GST-RBD/RA beads, cell lysis and precipitation of GTP-bound RAS were described elsewhere [1]. GST-$NF1^{333}$ beads were prepared likewise. After SDS PAGE and transfer

to polyvinylidene difluoride (PVDF) membranes, total and precipitated (active) HA-tagged HRAS was detected using peroxidase-conjugated rat monoclonal anti-HA antibody (Roche, Mannheim, Germany; clone 3F10; 1:5,000 dilution). For loading control, membranes were incubated with mouse anti-glyceraldehyde-3-phosphate dehydrogenase (GAPDH) (Abcam, Cambridge, UK; no. ab8245, 1:5,000 dilution), followed by peroxidase-coupled secondary anti-mouse antibody (Amersham Pharmacia Biotech, Freiburg, Germany; no. NA9310; 1:8,000 dilution). Data shown are representative of three independent experiments (**Figure 2**).

Immunoblotting

Twenty-four hours after transfection, cells were cultured as specified, washed with PBS and scraped off in modified radioimmunoprecipitation assay buffer (50 mM Tris-HCl, pH 8.0; 150 mM NaCl; 1% Nonidet P-40; 0.5% sodiumdeoxycholate; 0.1% SDS; 1 mM phenylmethylsulfonyl fluoride; 1 mM Na3VO4; 10 mM NaF; 1 Complete Mini protein inhibitor cocktail tablet [Roche] per 10 ml). Cellular biochemical reactions were stopped by freezing lysates in liquid nitrogen. After thawing on ice, cell debris was removed, solutions were supplemented with sample buffer, and proteins were separated on SDS-polyacrylamide gels and transferred to PVDF membranes. Following blocking (20 mM Tris-HCl, pH 7.4; 150 mM NaCl; 0.1% Tween-20; 4% non-fat dry milk) and washing (20 mM Tris-HCl, pH 7.4; 150 mM NaCl; 0.1% Tween-20), membranes were incubated in primary antibody solution (20 mM Tris-HCl, pH 7.4; 150 mM NaCl; 0.1% Tween-20; 5% BSA or 0.5% non-fat dry milk) containing the appropriate antibodies. Rabbit polyclonal antibodies against MEK1/2 (Cell Signaling Tech., Danvers, MA; no. 9122; 1:1000 dilution), phospho-MEK1/2 (Ser217/221) (Cell Signaling Tech.; no. 9121; 1:1000 dilution), p44/42 MAP kinase (ERK1/2) (Cell Signaling Tech.; no. 9102, 1:1000 dilution), phospho-p44/42 MAP kinase (ERK1/2) (Thr202/Tyr204) (Cell Signaling Tech.; no. 9101, 1:1000 dilution), Akt1/2/3 (Cell Signaling Tech.; no. 9272; 1:1000 dilution), and phospho-Akt1/2/3 (Ser473) (Cell Signaling Tech.; no. 9271; 1:1000 dilution) were used. Membranes were washed and incubated with peroxidase-coupled secondary anti-rabbit antibody (Amersham Pharmacia Biotech; no. NA9340V; 1:8000 dilution) or peroxidase-conjugated GST Tag monoclonal antibody (MA4-004, Invitrogen, Thermo Fisher Scientific,

Inc., Waltham, MA, USA). After final washing, immunoreactive proteins were visualized using the Immobilon Western Chemiluminescent HRP Substrate (Millipore, Schwalbach, Germany). Data shown are representative of three independent experiments (**Figures 2**, **S1**, **S2** and **S3**).

Statistical analysis

Signals on autoradiographs from three independent experiments were quantified by densitometric analysis using the ImageJ software (NIH; rsb.info.nih.gov/ij/index.html). Levels of active HA-HRAS, phosphorylated MEK1/2, ERK1/2 and AKT were normalized relative to levels of total HA-HRAS, MEK1/2, ERK1/2 and AKT, respectively. Maximum HA-HRAS activation levels and maximum MEK1/2, ERK1/2 and AKT phosphorylation levels were considered 100% for each stimulation series of cells expressing the indicated HRAS variants. A two-tailed unpaired Student's t-test was used to determine the significance of the difference between cells overexpressing different HRAS variants. Values are presented as the mean ± standard deviation and were considered significant at P-value < 0.05.

Genetic testing

Details on array-CGH and exome sequencing are available on request.

SUPPORTING RESULTS

Clinical summaries (extended versions)

Patient 1. The pregnancy was complicated by maternal gestational diabetes, polyhydramnios, fetal overgrowth and lower segment caesarean section at 37 weeks gestation. The birth weight of 4,510 g was above the 97th centile, length of 50 cm was on the 75th centile, and head circumference of 41 cm was greater than the 98th centile (**Table 1**). APGAR scores were 2, 6 and 7 at 1, 5 and 10 minutes, respectively. The baby was admitted to the neonatal intensive care on day 1 for suspected skeletal dysplasia because of apparent limb shortening, marked varus posture of wrist joints, adducted thumbs, elbow contractures, rocker bottom feet, extended knees, hip dysplasia and macrocephaly (**Figure 1A**). Known skeletal dysplasias were excluded clinically on review by clinical geneticist, and by limb and spine X-rays. The boy was discharged at 2 weeks old but re-admitted at 5 weeks old to paediatric intensive care for recurrent severe obstructive apneic episodes with cyanosis, loss of responsiveness, seizures and choking episodes during feeding. Pharyngeal and laryngeal obstruction required supraglottoplasty at age 6 weeks old and recurrent apneic episodes and aspiration required permanent tracheostomy at 8 weeks old. Dysfunctional swallowing and feeding difficulties were noted. Weight gains improved with small frequent percutaneous endoscopic gastrostomy (PEG) feeds. Focal seizures continued after stabilization of the airway with episodes of deviation of the eyes to one side, twitching of the contralateral hand and lip smacking and were treated with trials of levetiracetam and phenytoin, and topirimate, which was ceased because of metabolic acidosis and replaced by phenobarbital. Status epilepticus complicated an infection at the PEG site at 3 months old, requiring trials of sodium valproate and midazolam infusion, followed after stabilization by long-term clobazam, stiripentol and ketogenic diet with selenomethionine and carnitine supplementation. Seizure frequency reduced to one daily after resolution of the infection. There was increased extra axial cerebrospinal fluid (CSF) on magnetic resonance imaging (MRI) scan and electroencephalogram (EEGs) showed multiple foci of epileptiform activity on one occasion. At this point, genetic testing was initiated: High-resolution 850 K SNP chromosome microarray was normal. Trio exome sequencing identified two *de novo* variants,

c.466T>C p.(Phe156Leu) in *HRAS* (NM_005343.4) and c.4907G>C p.(Arg1636Pro) in *SCN1A* (NM_001165963.4). Both variants were considered likely pathogenic according to the ACMG/AMP guidelines [2]. Moreover, the *HRAS* c.466T>C variant has been classified to be pathogenic in the ClinVar database (Variation ID 1198752, ncbi.nlm.nih.gov/clinvar) [3]. Phenytoin was ceased after this result was available [4], and abdominal ultrasound detected no malignancy. The patient was following visually, smiling and breathing normally after removal of his tracheostomy at 9 months old. He had generalized developmental delay, was not able to roll or sit with marked head lag. Deep palmar and plantar creases were noted. Wrist, hand, hip, knee and foot contractures had improved with physiotherapy, serial splinting and occupational therapy. At 2 2/3 years, weight of 10 kg and length of 79.1 cm were below the 3rd centile and head circumference of 52 cm was greater than the 95th centile. Cardiac arrhythmia (ectipic beats) were documented; seizures persisted and are not well controlled.

Patient 2 was recently mentioned [5]. This boy weighed 3,508 g and had a head circumference of 36 cm (both 50th centile) after a normal vaginal delivery at term and a pregnancy complicated by polyhydramnios (**Table 1**). There was no relevant family history. APGAR scores were 9 and 10 at 1 and 5 minutes, respectively. Bilious vomiting, hypoglycemia, macroglossia and poor feeding were noted on day 1 of life. Daily episodes of hypoglycemia were associated with hyperinsulinism, treated with diazoxide and chlorothiazide. Severe hypoglycemia persisted with bolus feeds and continuous feeds were given via gastrostomy, with elemental formula due to cow's milk protein intolerance. Laryngomalacia was diagnosed by endoscopy and echocardiography identified hypertrophy of the ventricular myocardium and mild thickening of the pulmonary valve. The head turned to the right for preference but no sternomastoid fibrosis, other orthopedic or neurological explanation was identified. Metabolic and endocrinological investigations at 3 months old did not yield a diagnosis for the patient's atypical hyperinsulinism. Glucose levels were unresponsive to octreotide, were more stable on diazoxide and improved with prednisone suggesting increased insulin sensitivity in addition to hyperinsulinism. Subtotal (95%) pancreatectomy was required at 8 months old. During a 5 months hospitalization hypertrophic cardiomyopathy (HCM) and severe gastroesophageal reflux were

diagnosed. The boy has subtle CS facial features with a prominent forehead, long philtrum and full cheeks (**Figure 1B**). He has tight Achilles tendons and shows gait abnormalities. An abnormal spine profile was suspected by the patient's mother. Dermatologic findings included thickened skin on elbows, excessive sweating, and sensitive skin. The patient has a mild ventriculomegaly and moderate global developmental delay. Unilateral strabismus (esotropia) of the right eye was noted. At 6 2/3 yrs old hypoglycaemia episodes are still significant with several low glucose episodes repeatedly per day caused by any form of excitement or stimulation. Trio exome sequencing identified the *de novo HRAS* c.466C>T p.(Phe156Leu) variant.

Functional characterization

To analyze binding between HRAS and the RAS-specific NF1 GAP we pulled down HRAS protein variants by using the GAP-related domain of NF1 ($NF1^{333}$). We co-precipitated $HA\text{-}HRAS^{WT}$ together with GST-$NF1^{333}$ under any culture condition tested, likely reflecting the fraction of active $HA\text{-}HRAS^{WT}$ molecules in the lysates (**Figure S1**). Constitutively active $HA\text{-}HRAS^{Gly12Ser}$ and $HA\text{-}HRAS^{Gly12Val}$, but not the dominant negative variant $HA\text{-}HRAS^{Ser17Asn}$, strongly co-precipitated with GST-$NF1^{333}$ (**Figure S1**). The amount of co-precipitated $HA\text{-}HRAS^{Phe156Leu}$ was moderately increased compared to that derived from cells expressing $HA\text{-}HRAS^{WT}$; however, this increase was less pronounced than observed for $HA\text{-}HRAS^{Gly12Ser}$ and $HA\text{-}HRAS^{Gly12Val}$ (**Figure S1**). These results suggest that p.Phe156Leu does not negatively interfere with NF1 GAP binding.

To gain insight into consequences of p.Phe156Leu on signal traffic, we measured levels of phosphorylated AKT in cells expressing HRAS variants. AKT phosphorylation was only marginally increased in HEK293T cells expressing $HA\text{-}HRAS^{Phe156Leu}$, $HA\text{-}HRAS^{Gly12Val}$ or $HA\text{-}HRAS^{Gly12Ser}$ compared to $HA\text{-}HRAS^{WT}$ expressing cells; this weak AKT stimulation was detectable in serum-deprived and EGF stimulated cells but not under serum-saturated conditions (**Figure S2**). AKT signaling is very robust in HEK293T cells and dependence on HRAS as well as responsiveness to serum factors are limited in this cell line. Consequently, we verified these result by using another cell line, MCF-7 cells, and we found

that expression of HA-HRAS^{Phe156Leu}, HA-HRAS^{Gly12Val} or HA-HRAS^{Gly12Ser} induced stronger AKT phosphorylation than HA-HRAS^{WT} (**Figure 2B**).

Impaired signaling dynamics rather than a simple static hyperactivation of RAS-dependent signaling may underlie the development of CS [6,7]. We compared the intensity of EGF-induced HRAS downstream signaling in HEK293T cells over time. EGF stimulation induced a strong ERK1/2 phosphorylation response in HA-HRAS^{WT} cells after 5 minutes followed by signal decrease at 15 to 30 minutes after EGF addition (**Figures S3**, **2C**). In contrast, in cells expressing HA-HRAS^{Phe156Leu} or HA-HRAS^{Gly12Val} basal ERK1/2 phosphorylation (0 minutes EGF) was enhanced with little further increase (**Figures S3**, **2C**). Similarly, AKT phosphorylation was slightly stimulated upon EGF treatment in cells expressing HA-HRAS^{WT} but not or only marginally in cells expressing HA-HRAS^{Gly12Val} and HA-HRAS^{Phe156Leu}, respectively (**Figures S3**, **2C**).

SUPPORTING DISCUSSION

Phenotype associated with *HRAS* c.466T>C p.(Phe156Leu)

Although we only describe two patients with the p.Phe156Leu variant, it seems that there is a phenotypic variability regarding the pronounced musculoskeletal manifestation in patient 1 and the cardiovascular features and severe hypoglycemia in patient 2 (**Table 1**). In line with this, a variable clinical manifestation has also been reported for other rare *HRAS* variants [8-11].

Functional consequences of HRAS p.Phe156Leu

The germline variant p.Phe156Leu has also been identified in KRAS of patients with RASopathy [12,13]. Detailed functional characterization of $KRAS^{Phe156Leu}$ revealed (**i**) a marked increase in nucleotide exchange, (**ii**) a considerable reduction in GAP-stimulated GTPase rates, (**iii**) reduced binding affinities with RAF1 and RALGDS, (**iv**) increased phosphorylation levels of downstream signaling proteins, and (**v**) induction of cytokine-dependent hematopoietic progenitor colony growth [14,15]. Of note, p.Phe156Leu did not interfere with HRAS effector and NF1 GAP binding in our experiments (**Figure S1**). This may be caused by different sensitivities of applied assays or by the different nature of the analyzed samples: we used total cell lysates for protein precipitation assays, whereas others used recombinant proteins [14,15].

SUPPORTING FIGURES

Figure S1

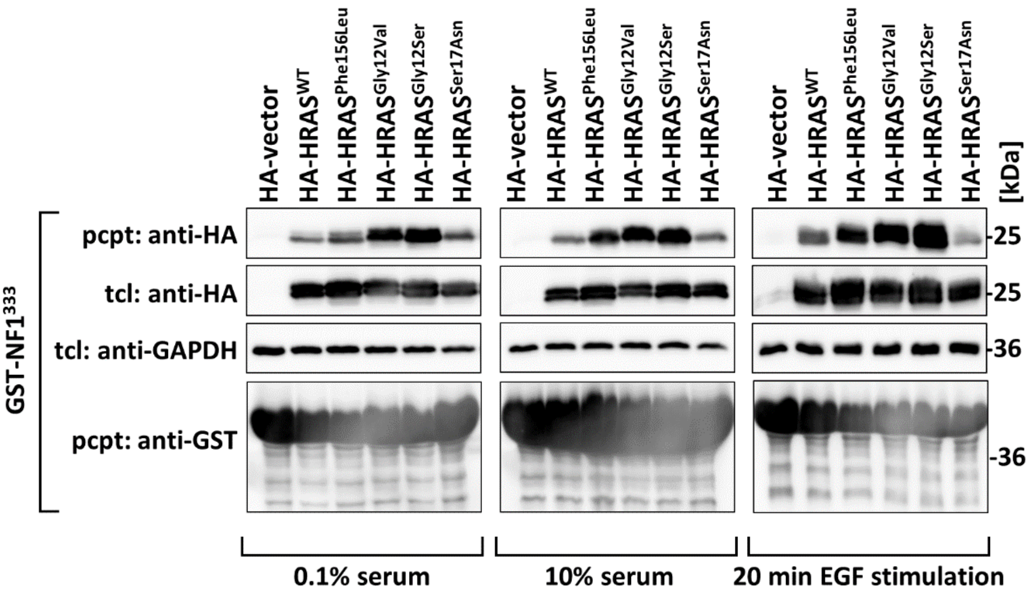

Figure S1. **p.Phe156Leu moderately increases co-precipitation efficiency with NF1.** HRAS variants were transiently expressed in HEK293T cells under serum-deprived (0.1% serum), basal (10% serum) and stimulated (10 ng/ml EGF for 20 min) conditions. HA-protein variants were precipitated from cell extracts by using the GST-fused, GAP-related domain of NF1 ($NF1^{333}$) and subjected to immunoblotting. Representative blots from 3 independent experiments (n = 3) are shown. Pcpt, precipitates; tcl, total cell lysates; NF1, neurofibromin 1; HA, hemagglutinin; GST, glutathione S-transferase.

Figure S2

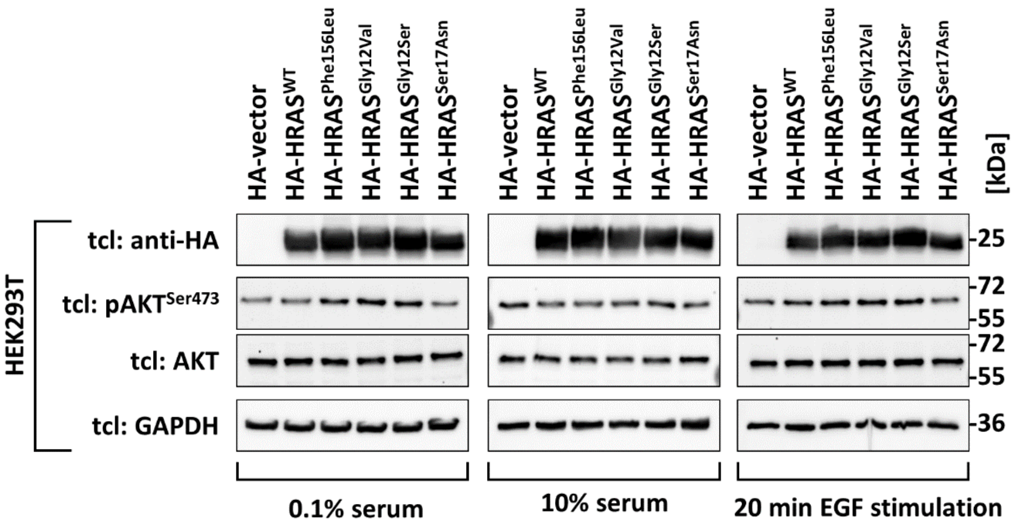

Figure S2. Consequences of HRAS p.Phe156Leu on AKT^{Ser473} phosphorylation in HEK293T cells. HRAS variants were transiently expressed in HEK293T cells. For control, cells were transfected with empty vector. Cells were cultured under serum-starved condition (0.1% serum), normal growth condition (10% serum), or serum-starved condition followed by 20 min stimulation with EGF (10 ng/ml EGF stimulation). Total cell lysates (tcl) were subjected to immunoblotting as indicated; n = 3.

Figure S3

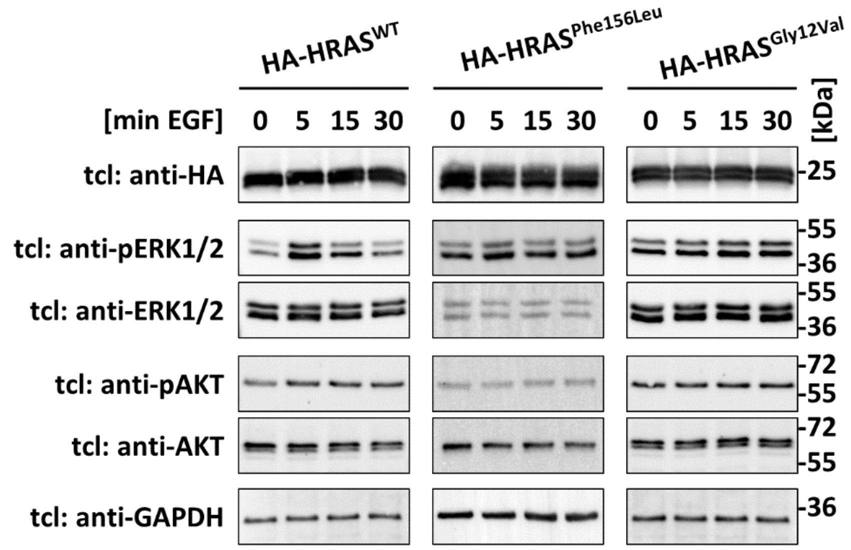

Figure S3. Expression of HRAS^{Phe156Leu} impairs epidermal growth factor sensitivity. HEK293T cells transiently expressing HRAS^{WT}, HRAS^{Gly12Val} or HRAS^{Phe156Leu} were stimulated with EGF for various times (5, 15, 30 min) or left untreated (0 min). Total cell lysates (tcl) were subjected to immunoblotting using specific antibodies as indicated; n = 3.

SUPPORTING REFERENCES

1. Rosenberger G, Meien S, Kutsche K Oncogenic HRAS mutations cause prolonged PI3K signaling in response to epidermal growth factor in fibroblasts of patients with Costello syndrome. Hum Mutat 2009;30:352-362.
2. Richards S, Aziz N, Bale S, Bick D, Das S, Gastier-Foster J et al. Standards and guidelines for the interpretation of sequence variants: a joint consensus recommendation of the American College of Medical Genetics and Genomics and the Association for Molecular Pathology. Genet Med 2015;17:405-424.
3. Landrum MJ, Lee JM, Benson M, Brown GR, Chao C, Chitipiralla S et al. ClinVar: improving access to variant interpretations and supporting evidence. Nucleic acids research 2018;46:D1062-D1067.
4. Miller IO, Sotero de Menezes MA: SCN1A Seizure Disorders; in: Adam MP, Ardinger HH, Pagon RA *et al.* (eds): *GeneReviews((R))*. Seattle (WA), 1993.
5. Gripp KW, Morse LA, Axelrad M, Chatfield KC, Chidekel A, Dobyns W et al. Costello syndrome: Clinical phenotype, genotype, and management guidelines. American journal of medical genetics Part A 2019;179:1725-1744.
6. Lorenz S, Lissewski C, Simsek-Kiper PO, Alanay Y, Boduroglu K, Zenker M et al. Functional analysis of a duplication (p.E63_D69dup) in the switch II region of HRAS: new aspects of the molecular pathogenesis underlying Costello syndrome. Hum Mol Genet 2013;22:1643-1653.
7. Gripp KW, Kolbe V, Brandenstein LI, Rosenberger G Attenuated phenotype of Costello syndrome and early death in a patient with an HRAS mutation (c.179G>T; p.Gly60Val) affecting signalling dynamics. Clinical genetics 2017;92:332-337.
8. Gripp KW, Hopkins E, Sol-Church K, Stabley DL, Axelrad ME, Doyle D et al. Phenotypic analysis of individuals with Costello syndrome due to HRAS p.G13C. Am J Med Genet A 2011;155A:706-716.

9. Gripp KW, Innes AM, Axelrad ME, Gillan TL, Parboosingh JS, Davies C et al. Costello syndrome associated with novel germline HRAS mutations: an attenuated phenotype? American journal of medical genetics Part A 2008;146A:683-690.

10. Gripp KW, Hopkins E, Serrano A, Leonard NJ, Stabley DL, Sol-Church K Transmission of the Rare HRAS Mutation (c. 173C>T; p.T58I) Further Illustrates its Attenuated Phenotype. American journal of medical genetics Part A 2012;158A:1095-1101.

11. Gripp KW, Sol-Church K, Smpokou P, Graham GE, Stevenson DA, Hanson H et al. An attenuated phenotype of Costello syndrome in three unrelated individuals with a HRAS c.179G>A (p.Gly60Asp) mutation correlates with uncommon functional consequences. American journal of medical genetics Part A 2015;167A:2085-2097.

12. Zenker M, Lehmann K, Schulz AL, Barth H, Hansmann D, Koenig R et al. Expansion of the genotypic and phenotypic spectrum in patients with KRAS germline mutations. J Med Genet 2007;44:131-135.

13. Sovik O, Schubbert S, Houge G, Steine SJ, Norgard G, Engelsen B et al. De novo HRAS and KRAS mutations in two siblings with short stature and neuro-cardio-facio-cutaneous features. J Med Genet 2007;44:e84.

14. Gremer L, Merbitz-Zahradnik T, Dvorsky R, Cirstea IC, Kratz CP, Zenker M et al. Germline KRAS mutations cause aberrant biochemical and physical properties leading to developmental disorders. Hum Mutat 2010;32:33-43.

15. Schubbert S, Bollag G, Lyubynska N, Nguyen H, Kratz CP, Zenker M et al. Biochemical and functional characterization of germ line KRAS mutations. Mol Cell Biol 2007;27:7765-7770.
